# Potential Impact of Rapid Blood Culture Testing for Gram-Positive Bacteremia in Japan with the Verigene Gram-Positive Blood Culture Test

**DOI:** 10.1155/2017/4896791

**Published:** 2017-02-20

**Authors:** Ken Kikuchi, Mari Matsuda, Shigekazu Iguchi, Tomonori Mizutani, Keiichi Hiramatsu, Michiru Tega-Ishii, Kaori Sansaka, Kenta Negishi, Kimie Shimada, Jun Umemura, Shigeyuki Notake, Hideji Yanagisawa, Hiroshi Takahashi, Reiko Yabusaki, Hideki Araoka, Akiko Yoneyama

**Affiliations:** ^1^Department of Infectious Diseases, Tokyo Women's Medical University, 8-1 Kawada-cho, Shinjuku-ku, Tokyo 162-8666, Japan; ^2^Sakakibara Heart Institute, 1-16-1 Asahimachi, Fuchu, Tokyo 183-0003, Japan; ^3^Department of Infection Control Science, Faculty of Medicine, Juntendo University, 2-1-1 Hongo, Bunkyo-ku, Tokyo 113-8421, Japan; ^4^Miroku Medical Laboratory Inc., 659-2 Innai, Saku, Nagano 384-2201, Japan; ^5^East-West Diagnostics/Theranostics, San Francisco, CA 94105, USA; ^6^Department of Infectious Diseases, Toranomon Hospital, 2-2-2 Toranomon, Minato-ku, Tokyo 105-8470, Japan

## Abstract

*Background.* Early detection of Gram-positive bacteremia and timely appropriate antimicrobial therapy are required for decreasing patient mortality. The purpose of our study was to evaluate the performance of the Verigene Gram-positive blood culture assay (BC-GP) in two special healthcare settings and determine the potential impact of rapid blood culture testing for Gram-positive bacteremia within the Japanese healthcare delivery system. Furthermore, the study included simulated blood cultures, which included a library of well-characterized methicillin-resistant* Staphylococcus aureus* (MRSA) and vancomycin-resistant enterococci (VRE) isolates reflecting different geographical regions in Japan.* Methods.* A total 347 BC-GP assays were performed on clinical and simulated blood cultures. BC-GP results were compared to results obtained by reference methods for genus/species identification and detection of resistance genes using molecular and MALDI-TOF MS methodologies.* Results.* For identification and detection of resistance genes at two clinical sites and simulated blood cultures, overall concordance of BC-GP with reference methods was 327/347 (94%). The time for identification and antimicrobial resistance detection by BC-GP was significantly shorter compared to routine testing especially at the cardiology hospital, which does not offer clinical microbiology services on weekends and holidays.* Conclusion.* BC-GP generated accurate identification and detection of resistance markers compared with routine laboratory methods for Gram-positive organisms in specialized clinical settings providing more rapid results than current routine testing.

## 1. Introduction

Gram-positive bacteria are the most predominant microorganisms associated with sepsis in healthcare settings and the most prevalent cause of bacteremia in patients with hematopoietic stem cell transplantation [1, 2]. Enterococcal bacteremia is associated with increased risk of mortality in patients with hematopoietic stem cell transplantation, irrespective of susceptibility to vancomycin [3, 4]. In cardiology units, infective endocarditis, infectious aneurysm, catheter-related bloodstream infections, or surgical site infections after cardiac surgeries are the major infections of which the most common causative microorganisms are Gram-positive cocci [5–10]. In both medical units, early detection of Gram-positives and resistant markers is very critical in managing patient care, antibiotic stewardship, and preventing spread of resistant microorganisms.

As early intervention with antimicrobial therapy is associated with improved prognosis with each hour of delay associated with increased mortality, rapid diagnosis is critical [11]. The Verigene Gram-positive blood culture assay (BC-GP) (Nanosphere, Inc., Northbrook, IL) is a sample to result microarray system for identification of common Gram-positive bacteria and major resistance markers directly from positive blood culture. Although a number of studies have previously evaluated BC-GP reporting performance ranging from 92 to 99% agreement with conventional methodology [12–16], one limitation of previous reports has been that many of the studies have been from the United States as well as countries outside of Japan with only one published report of limited scope in Japan [17].

Genetic variation among bacterial lineages circulating in different geographical regions of the world can affect the sensitivity of molecular assays based on oligonucleotide probes to detect organisms or resistance markers. Studies in Hong Kong and Belgium have reported lower BC-GP performance [18, 19].

The purpose of this study was to determine the potential impact on patient management and patient outcome of BC-GP in specialized hospitalized settings within the Japanese healthcare environment, which faces a number of challenges. An increasingly aging population and the accompanying burden of increasing overall healthcare costs have challenged the healthcare infrastructure. Despite methicillin-resistant* Staphylococcus aureus* (MRSA) accounting for over 90% of the hospital-acquired infections caused by resistant bacteria in Japan [20], outsourcing of clinical microbiology testing or no weekend coverage is common in many healthcare facilities as part of cost-containment. This paper represents the first comprehensive evaluation of BC-GP in Japan to validate its clinical performance. The simulated blood culture study includes a library of well-characterized healthcare-associated MRSA (HA-MRSA), community-acquired MRSA (CA-MRSA), and vancomycin-resistant enterococci (VRE) strains circulating in Japan.

## 2. Methods

BC-GP was evaluated at Toranomon Hospital (TH) and Sakakibara Heart Institute (SHI), in accordance with site-specific institutional review board approved study protocols, during June 26, 2012, to March 6, 2013. TH is an 1168-bed general teaching hospital with a 123-bed hematological care unit for hematopoietic stem cell transplantation performing 140 to 160 hematopoietic stem cell transplants per year. The microbiology laboratory at the hospital operates daily during day hours. SHI is a 320-bed teaching hospital specializing in cardiovascular diseases with a caseload of over 1,500 open-heart surgeries per year. The microbiology laboratory in the hospital is operated by an outside commercial reference laboratory. The microbiology laboratory operates during the day shift on weekdays and closed on weekends and holidays.

Blood cultures were performed at SHI using BacT/ALERT FA bottles and monitoring with BacT/ALERT 3D (bioMérieux, Marcy l'Etoile, France). TH used BACTEC Plus bottles and monitoring with BACTEC 9240 and FX (Becton Dickinson, Franklin Lakes, and NJ). Only one positive blood culture bottle containing Gram-positive cocci or bacilli per patient was included in the study. Two ml of positive blood culture medium was stored at −85C for retesting.

Routine microbiological identification and susceptibility testing of isolates were performed using conventional identification tests such as bile solubility, optochin disk susceptibility, and the MicroScan WalkAway system (Beckman Coulter, Pasadena, CA) at TH and the Vitek 2 system (bioMérieux, Marcy l'Etoile, France) at SHI. Cefoxitin screening for methicillin resistance was performed according to CLSI guidelines [21]. A latex agglutination test for detection of penicillin-binding protein PBP2a was also utilized [22].

BC-GP testing was performed on positive blood culture showing Gram-positive organisms according to the manufacturer's instructions. Briefly, a well-mixed 350 *μ*l sample of the blood culture media was pipetted into the sample well of the BC-GP nucleic extraction tray, placed onto the Verigene Processor* SP* for processing and analysis by the Verigene Reader.

An assessment was performed to determine the difference in time between reporting of results using BC-GP and culture-based identification and antimicrobial susceptibility results for 139 positive blood culture broths. For culture-based results, the time required until generation of the final report was the time between the Gram stain reading and entering of final identification and susceptibility results into the laboratory information system. For BC-GP, the time to result was the time between the Gram stain reading and entering of BC-GP results into the laboratory information system.

A challenge set of 208 simulated blood cultures was constructed using type, reference strains, and clinical strains from different geographical regions in Japan to evaluate BC-GP. The clinical strains included organisms that presented challenges for commercial identification systems in previous clinical studies at TH and SHI. In addition, a library of well-characterized HA-MRSA, CA-MRSA, and VRE strains from Japan was also tested. Simulated blood culture studies were performed at Miroku Medical Laboratory (Saku City, Nagano Prefecture, Japan). Two hundred and eight strains were adjusted to a turbidity of approximately 100 CFU/ml in sterile saline. Three hundred *μ*l was inoculated into BACTEC Plus Aerobic/F bottles containing 8 to 10 ml of human whole blood (blood type O, Tennessee Blood Services, Memphis, TN) for a final inoculum of 30 CFU/bottle. A BACTEC Plus Anaerobic/F bottle was also inoculated for* S. pneumoniae* and the* S. anginosus* group. Each bottle was incubated in the BACTEC system until a positive signal was generated. If BC-GP generated negative results, 11-fold dilution of the blood culture medium using sterile distilled water was retested.

Each of the positive blood culture isolates at the two hospital sites was stored in 10% skim milk (Difco) at −85C. Species identification was confirmed using matrix-assisted laser desorption ionization-time of flight mass spectrometry (MALDI-TOF MS) (Microflex LT with Biotyper ver. 3.0 software; Bruker Daltonik GmbH, Bremen, Germany) for all strains [23]. If the organism was not identified to the species level by MALDI-TOF MS (score value < 2.0) or identified as* Micrococcus, Listeria, Staphylococcus* other than* S. aureus, Streptococcus* other than* S. pyogenes,* and* S. agalactiae*, confirmatory testing by PCR-direct sequencing of 16S rDNA or* sodA* was performed at Juntendo University or Tokyo Women's Medical University [24–26]. Specific-PCR for detecting* mecA* [27] in all staphylococci and* vanA*,* vanB* in all enterococci was performed [28]. The methods used for SCC*mec *typing of community-acquired or healthcare-associated MRSA used to characterized strains have been previously described [29, 30].

Concordance was determined in comparison to results of the reference methods. There was agreement if BC-GP target detection agreed with the reference method at either the genus or the species level. The ninety-five percent confidence intervals (95% CI) and the paired *t*-test were determined using GraphPad StatMate (GraphPad Software Inc., San Diego, CA).

## 3. Results

In this study, the overall identification agreement between BC-GP and the reference method was 327/347 (94%) for prospective blood cultures at two clinical sites and simulated blood cultures. The combined agreement between PCR and BC-GP for* mecA *detection was 71/73 (97%) for prospective blood cultures and simulated blood cultures.

The combined identification accuracy from both hospital sites was 129/139 (93%). For monomicrobial cultures, the identification accuracy was 121/124 (98%). Agreement between PCR and BC-GP for* mecA* positivity was 51/53 (96%).  Table 1 shows the results from TH. Overall, 96/104 (92%) organisms were correctly identified by BC-GP to the species or genus level including detection of resistance genes. As shown in Table 2, total agreement of BC-GP with the reference method at SHI was 33/35 (94%) organisms.

BC-GP reports the presence of* mecA *for only* S. aureus* and* S. epidermidis*. In this study, of the 102 staphylococcal strains, 72 were either* S. aureus *or* S. epidermidis*. Discordant results were due to undetectable* mecA S. epidermidis *organisms in polymicrobial cultures containing both* mecA *positive and* mecA* negative* S. epidermidis*. Of the 30* Staphylococcus* spp., other than* S. epidermidis* and* S. aureus,* 21 (70%) were positive for* mecA*, including 2* S. lugdunensis*, which could not be reported as* mecA* positive by BC-GP.

Table 3 shows the difference in time between the generation of BC-GP results and culture-based final identification and antimicrobial susceptibility results at both hospitals. BC-GP results were available at a mean of 28.2 to 51.0 hours before culture-based final identification and susceptibility results at TH. At SHI, BC-GP generated results at a mean of 34.5 to 196.6 hours earlier. In comparing the time to final culture-based identification and susceptibility results at TH and SHI, with the exception of* S. aureus*, results for* S. epidermidis* and coagulase-negative staphylococci other than* S. epidermidis*, enterococci, and streptococci required significantly (*P* < 0.05) longer time at SHI (83.3, 123.6, 159.1, and 199.1 hours, resp.) compared to TH (40.8, 53.9, 36.1, and 53.5 hours, resp.).

Using simulated Gram-positive blood cultures, BC-GP correctly identified 198/208 (95%) of the organisms (Table 4). Six streptococci (3%) were either incorrectly identified or identified at the genus level only by BC-GP. With respect to the 4 BC-GP false-negative blood culture bottles, 1* S. pyogenes* was correctly identified and 1* S. mitis *generated a positive* Streptococcus *genus/*S. pneumoniae* signal following 11-fold dilution of the blood culture medium. The* mecA *gene was detected in 20/20 (100%) MRSA organisms representing community-acquired (SCC*mec* type IIa, IV, and V) and healthcare-associated (SCC*mec* type I, IIb, III, and nontypeable) strains by BC-GP. BC-GP detected 14/14 (100%)* vanA* and 20/20 (100%)* vanB* genes in well-characterized VRE strains from previous studies in Japan.

## 4. Discussion

The performance of BC-GP observed in our study was similar to previous reports [12–16, 31–33]. As clinical microbiology services are outsourced or not operating during off-shifts during weekdays and closed on weekends and holidays in many hospitals in Japan, rapid diagnostic testing has significant potential to impact patient care by dramatically decreasing the time to organism identification and antimicrobial susceptibility results.

BC-GP detects* mecA* in all staphylococci based on measurement of signal intensity; however, reporting is restricted to* S. aureus *and* S. epidermidis* based on an algorithm in which* mecA *is only reported when* S. aureus* or* S. epidermidis *is detected by BC-GP. Future versions of BC-GP should consider modification of the algorithm to allow reporting of* mecA* detection for staphylococci other than* S. aureus* or* S. epidermidis* as 70% of the 30 non-*S. aureus* and* S. epidermidis* strains in our study were methicillin-resistant. Although* S. epidermidis* is the major coagulase-negative staphylococcal pathogen, other staphylococci such as* S. lugdunensis* and* S. haemolyticus* are important pathogens in the healthcare environment [34, 35].

Polymicrobial positive blood cultures generated the majority of the discordance. In contrast, the performance of BC-GP was 121/124 (98%) in monomicrobial clinical blood cultures and 198/208 (95%) in simulated blood cultures. In this study, polymicrobial blood cultures accounted for 14/106 (13%) and 3/33 (9%), respectively, of the positive blood cultures at TH and SH. Combined, polymicrobial cultures represented 17/139 (12%) of the prospective clinical blood cultures which is consistent with previous studies reporting 6 to 20% of all bloodstream infections to be polymicrobial [36–38]. BC-GP correctly identified all of the organisms in 12/17 (70%) of polymicrobial cultures. In previous BC-GP studies, the correct identification rates in polymicrobial cultures ranged from 57 to 86% [14–16, 31, 32]. As misleading information can affect clinical diagnosis, resulting in inappropriate selection of antimicrobial agents, there is a need to understand the limitations of BC-GP.

An additional concern with polymicrobial cultures is the clinical interpretation of* mecA* detection and staphylococcal detection. In our 17-polymicrobial cultures, 5 samples yielded 2 or 3 staphylococcal strains with or without* mecA*. This may lead to unnecessary use of vancomycin or underestimating infection caused by methicillin-resistant staphylococci. Repeating BC-GP on another set of blood cultures may lessen the risk of this problem. For monomicrobial cultures or simulated cultures, all of the discrepancies were observed with streptococci except for 1* S. caprae* strain. BC-GP misidentification for streptococci included 2* S. mitis* identified as* S. pneumoniae*, no detection of 2* S. pneumoniae*, 2* S. anginosus* group, and 2* S. pyogenes. *Previous reports have also reported similar results for* S. mitis, S. oralis,* and* S. pneumoniae* [14–16, 39]. As genetic relatedness among* S. mitis, S. oralis, *and* S. pneumoniae* is well known based on >99% homology of 16S rRNA gene sequences [24], BC-GP results for* S. pneumoniae* or* Streptococcus *with alpha-hemolysis without any positive species-specific signals should be carefully interpreted and confirmed by conventional methods such as optochin sensitivity or the bile solubility test. Interestingly, 11-fold dilution of the original blood culture medium can lead to detection of the* Streptococcus* signal and species signal (Table 4). A range of detection depending on the organism by BC-GP has been reported [40].

The major benefit of utilizing BC-GP is the earlier time for reporting identification and resistance determinants from positive blood cultures allowing for earlier selection of appropriate antimicrobial therapy and implementation of infection control measures such as isolation and contact precaution [39, 41]. This has the potential of making a significant impact in the Japanese healthcare delivery system. The difference in time between BC-GP results and final culture-based identification and susceptibility results shown in Table 3 at TH is consistent with previous reports [14–16, 18, 32, 41]. On the other hand, earlier results of 80.7 to 196.6 hours for organisms other than* S. aureus *using BC-GP at SHI reflects the unavailability of clinical microbiology services during weekends and holidays. As clinical microbiology services are outsourced in many hospitals in Japan or limited to one shift during weekdays or not offered during weekends, the potential cost-benefits of retaining blood culture services in hospitals are significant. Furthermore, training of laboratory personnel in the general laboratory to recognize Gram-positive cocci and bacilli will allow BC-GP testing over the weekend as well as evenings/nights. BC-GP provides the opportunity for hospitals to retain in-house a very critical laboratory service.

In conclusion, BC-GP provided accurate identification and detection of resistance markers compared with routine culture-based laboratory methods for Gram-positive organisms including CA-MRSA, HA-MRSA, and VRE strains circulating in Japan. Minimizing the time to optimizing antimicrobial therapy using BC-GP may contribute to reduced costs and improved patient care. In 2016, BC-GP received regulatory approval in Japan becoming the first multitarget molecular test for positive blood cultures approved as an in vitro diagnostic device to aid in the diagnosis of bacterial bloodstream infections. Additional studies will be necessary to validate the cost-effectiveness of BC-GP within the context of the Japanese healthcare delivery system.

## Figures and Tables

**Table 1:** Performance of the BC-GP assay at Toranomon Hospital.

Organism	BC-GP (total)				BC-GP (monomicrobial cultures)			
	Number of organisms	Number (%) of isolates			Number of organisms	Number (%) of isolates		
		Correctly identified	Not detected	Incorrectly identified		Correctly identified	Not detected	Incorrectly identified
*Staphylococcus*	78	73 (94)	4 (5)	1 (1)	71	70 (99)	1 (2)	
*S. aureus*	16	16 (100)			16	16 (100)		
Methicillin-sensitive	9	9 (100)			9	9 (100)		
Methicillin-resistant	7	7 (100)			7	7 (100)		
*S. epidermidis*	37	34 (92)	2 (5)	1 (3)	34	34 (100)		
Methicillin-sensitive	2	1 (50)	1 (50)^a^		1	1 (100)		
Methicillin-resistant	35	33 (94)	1 (3)^b^	1 (3)^c^	33	33 (100)		
*S. lugdunensis*	1	1 (100)			1	1 (100)		
Other CNS	24	22 (92)	2 (8)		20	19 (95)	1 (6)	
*S. caprae*	9	8 (89)	1 (11)		7	6 (67)	1 (33)	
*S. hominis*	8	8 (100)			7	7 (100)		
*S. haemolyticus*	4	3 (75)	1 (25)^d^		3	3 (100)		
*S. capitis*	1	1 (100)			1	1 (100)		
*S. schleiferi*	1	1 (100)			1	1 (100)		
*S. simulans*	1	1 (100)			1	1 (100)		

*Streptococcus*	9	7 (78)	1 (11)	1 (11)	6	5 (83)		1 (27)
*S. agalactiae*	1	1 (100)			1	1 (100)		
*S. anginosus *group	1	1 (100)						
*S. constellatus*	1	1 (100)^e^						
Other streptococci	7	5 (72)	1 (14)	1 (14)	5	4 (80)		
*S. mitis*	2	1 (50)		1 (50)^f^	2	1 (50)		1 (50)^f^
*S. infantis*	2	1 (50)	1 (50)^g^		1	1 (100)		
*S. tigurinus*	2	2 (100)			1	1 (100)		
*S. oralis*	1	1 (100)			1	1 (100)		

*Enterococcus*	17	16 (94)	1 (6)		16	16 (100)		
*E. faecalis*	2	1 (50)	1 (50)^h^		1	1 (100)		
Vancomycin-sensitive	2	1 (50)	1 (50)		1	1 (100)		
*E. faecium*	15	15 (100)			15	15 (100)		
Vancomycin-sensitive	15	15 (100)			15	15 (100)		

Total	104	96 (92)	6 (6)	2 (2)	93	91 (98)	1 (1)	1 (1)

Other nontarget Gram-positives	15				10			
*Bacillus*	3				2			
*B. subtilis*	2				1			
*B. cereus*	1				1			
*Corynebacterium*	12				8			
*C. striatum*	9				5			
*C. jeikeium*	3				3			

Total isolates	119				103			

^a^Polymicrobial culture of methicillin-sensitive and methicillin-resistant *S. epidermidis.*

^b^Polymicrobial culture with *E. faecium.*

^c^Correctly identified as *Staphylococcus*, but not as *S. epidermidis, S. aureus,* or *S. lugdunensis.*

^d^Polymicrobial culture with *S. tigurinus.*

^e^“*S. anginosus *group” identified by the BC-GP assay is defined as “correctly identified” for each species.

^f^Identified as *S. pneumoniae.*

^g^Polymicrobial culture with methicillin-resistant *S. epidermidis.*

^h^Polymicrobial culture with *Escherichia coli.*

**Table 2:** Performance of the BC-GP assay at Sakakibara Heart Institute.

Organism	BC-GP (total)				BC-GP (monomicrobial cultures)			
	Number of organisms	Number (%) of isolates			Number of organisms	Number (%) of isolates		
		Correctly identified	Not detected	Incorrectly identified		Correctly identified	Not detected	Incorrectly identified
*Staphylococcus*	24	24 (100)			22	22 (100)		
*S. aureus*	7	7 (100)			7	7 (100)		
Methicillin-sensitive	6	6 (100)			6	6 (100)		
Methicillin-resistant	1	1 (100)			1	1 (100)		
*S. epidermidis*	12	12 (100)			11	11 (100)		
Methicillin-sensitive	2	2 (100)			2	2 (100)		
Methicillin-resistant	10	10 (100)			9	9 (100)		
*S. lugdunensis*	1	1 (100)			1	1 (100)		
Other CNS	4	4 (100)			3	3 (100)		
*S. hominis*	2	2 (100)			2	2 (100)		
*S. haemolyticus*	1	1 (100)						
*S. capitis*	1	1 (100)			1	1 (100)		

*Streptococcus*	8	8 (100)			8	7 (87)		1 (13)^b^
*S. pyogenes*	1	0 (0)		1 (100)^a^	1	0 (0)		1 (100)^b^
*S. agalactiae*	1	1 (100)			1	1 (100)		
*S. anginosus *group	2	2 (100)			2	2 (100)		
*S. anginosus*	2	2 (100)^b^			2	2 (100)^c^		
Other streptococci	4	4 (100)			4	4 (100)		
*S. oralis*	2	2 (100)			2	2 (100)		
*S. sanguinis*	1	1 (100)			1	1 (100)		
*S. parasanguinis*	1	1 (100)			1	1 (100)		
*Enterococcus*	2	1 (50)	1 (50)					
*E. faecalis*	1	0 (0)	1 (100)^c^					
Vancomycin-sensitive	1	0 (0)	1 (100)^c^					
*E. faecium*	1	1 (100)						
Vancomycin-sensitive	1	1 (100)						

*Listeria *spp.	1	1 (100)			1	1 (100)		

Total	35	33 (94)	1 (3)	1 (3)	31	30 (97)		1 (3)

Other nontarget Gram-positives	1				1			
*Corynebacterium striatum*	1				1			

Total isolates	36				32			

^a^Identified to the genus level, but not to the species level.

^b^“*S. anginosus *group” identified by the BC-GP assay is defined as “correctly identified” for each species.

^c^Polymicrobial culture with *S. epidermidis.*

**Table 3:** Difference in time to final identification and antimicrobial susceptibility report.

Organism	Sakakibara Heart Institute		Toranomon Hospital	
	Mean (h)	Range	Mean (h)	Range
*S. aureus*	34.5 (*P* < 0.05)	21.5–46.8	28.2 (*P* < 0.05)	19.6–47.0
*S. epidermidis*	80.7 (*P* < 0.05)	23.9–160.9	38.3 (*P* < 0.05)	21.6–72.5
Coagulase-negative staphylococci	121.1 (*P* < 0.05)	25.9–217.4	51.4 (*P* < 0.05)	21.4–72.2
*Enterococcus* spp.	156.6 (*P* < 0.05)	95.9–217.4	33.6 (*P* < 0.05)	23.4–47.7
*Streptococcus* spp.	196.6 (*P* < 0.05)	42.3–502.6	51.0 (*P* < 0.05)	23.4–69.2

^a^The difference in time between BC-GP result and final culture-based identification and susceptibility results.

**Table 4:** Detection of Gram-positive bacteria and resistance genes in simulated blood cultures by BC-GP.

Organism	Total no. of strains	No. (%) of isolates		
		correctly identified	not detected	incorrectly identified
*Staphylococcus*	54	54 (100)		
*S. aureus*	35	35 (100)		
Methicillin-sensitive, *mecA*−	15	15 (100)		
Methicillin-resistant, *mecA*+	20	20 (100)		
Health-care associated	10	10 (100)		
Community acquired	10	10 (100)		

*S. epidermidis*	1	1 (100)		
Methicillin-sensitive, *mecA*−	1	1 (100)		

*S. lugdunensis*	8	8 (100)		

Other CNS	10	10 (100)		
*S. hominis*	2	2 (100)		
*S. haemolyticus*	2	2 (100)		
*S. saprophyticus*	2	2 (100)		
*S. capitis*	1	1 (100)		
*S. cohnii*	1	1 (100)		
*S. warneri*	1	1 (100)		
*S. pseudintermedius*	1	1 (100)		

*Streptococcus*	87	77 (88)	4 (5)	6 (7)
*S. pyogenes*	9	8 (89)	1 (11)^a^	
*S. agalactiae*	8	8 (100)		
*S. dysgalactiae*	8	8 (100)		
*S. anginosus *group^b^	10	8 (80)		2 (20)
*S. anginosus*	3	2 (66)		1 (34)^c^
*S. constellatus*	4	3 (75)		1 (25)^c^
*S. intermedius*	3	3 (100)		
*S. pneumoniae*	22	20 (91)		2 (9)^d^
Other streptococci	30	25 (86)	3 (10)	2 (7)
*S. mitis*	12	9 (75)	1 (8)	2 (17)^e^
*S. oralis*	4	4 (100)		
*S. infantis*	2	2 (100)		
*S. mutans*	2	1 (50)	1 (50)	
*S. sobrinus*	1	0 (0)	1 (100)	
*S. sanguinis*	1	1 (100)		∖
*S. parasanguinis*	1	1 (100)		
*S. peroris*	1	1 (100)		
*S. australis*	1	1 (100)		
*S. tigurinus*	1	1 (100)		
*S. cristatus*	1	1 (100)		
*S. gordonii*	1	1 (100)		
*S. gallolyticus*	1	1 (100)		
*S. lutetiensis*	1	1 (100)		

*Enterococcus*	57	57 (100)		
*E. faecalis*	32	32 (100)		
Vancomycin-sensitive	18	18 (100)		
Vancomycin-resistant, *vanA*+	4	4 (100)		
Vancomycin-resistant,* vanB*+	10	10 (100)		
*E. faecium*	25	25 (100)		
Vancomycin-sensitive	5	5 (100)		
Vancomycin-resistant, *vanA*+	10	10 (100)		
Vancomycin-resistant,* vanB*+	10	10 (100)		

*Listeria *spp.	5	5 (100)		

*Micrococcus *spp.	5	5 (100)		

Total	208	198 (95)	4 (2)	6 (3)

^a^Not detected initially, but positive using 11-fold diluted blood culture sample.

^b^ *Streptococcus *sp. belonging to the *S. anginosus group *is identified as* S. anginosus *group by BC-GP.

^c^Positive signal for *Streptococcus*, but no signal for *S. anginosus* group.

^d^Positive signal for *Streptococcus*, but no signal for *S. pneumoniae.*

^e^Misidentified as *S. pneumoniae.*
